# Deep brain stimulation and suicide attempts in treatment-resistant patients: a case report and neuroethical analysis

**DOI:** 10.3389/fpsyt.2024.1398777

**Published:** 2024-06-26

**Authors:** Ambra D’Imperio, Marcello Ienca

**Affiliations:** ^1^ Département de Psychiatrie, Hôpitaux Universitaires de Genève, Geneva, Switzerland; ^2^ Institut für Geschichte und Ethik der Medizin, Technische Universität München, Munich, Germany; ^3^ College of Humanities, École Polytechnique Fédérale de Lausanne (EPFL), Lausanne, Switzerland

**Keywords:** DBS (deep brain stimulation), suicidality, neuroethical considerations, multiple system atrophy Parkinsonian predominant type, case report

## Abstract

This case presents the situation of a 66-year-old woman diagnosed with Multiple System Atrophy Parkinsonian Type who underwent deep brain stimulation (DBS) therapy and subsequently made two suicide attempts. Despite receiving treatment and extensive psychotherapy, her condition did not improve, leading to suicidal behavior over the course of a year. Notably, she held unrealistic beliefs about the effectiveness of DBS therapy, expressing dissatisfaction with its outcomes. Family dynamics were complex, with the patient concealing her psychological distress while coping with her worsening health condition. This severe distress culminated in two suicide attempts within a relatively short timeframe. Our psychiatric team promptly intervened, implementing a suicidality protocol and adjusting her medication regimen. Despite a documented prevalence of suicidal ideation and attempts post-DBS in the literature, the exact causes remain uncertain, with the suggested involvement of neuroimmune or neurological pathways. This case contributes to scientific understanding by shedding light on suicide attempts following ineffective DBS interventions, emphasizing the patient’s right to be informed about potential suicide risks and the possibility of assisted suicide through a neuroethical analysis. Therefore, our case underlines the importance of psychiatric evaluation and intervention in DBS patients to prevent further suicidality, focusing on a multidisciplinary approach tailored to the patient’s autonomy and neuroethical principles.

## 1 Introduction

Numerous studies have documented the occurrence of suicidal ideation and attempts following deep brain stimulation (DBS) procedures, with an incidence rate of 4% of suicidal ideation, 1% of attempted suicide, and 1% of suicide rate reported in the literature (1). Although the literature indicates a conspicuous occurrence of suicidal ideation and attempts post-DBS, the discussion regarding etiology remains nebulous: various hypotheses regarding the etiology of this phenomenon have been proposed, exploring potential pathways involving alterations in the patient’s will, particularly implicating the basal ganglia system and extrapyramidal fibers, defining a connectome valid for deliberate actions as suicide (2). Furthermore, the neurobiological explanation of this phenomenon has been examined, mainly focusing on the dopamine pathway and the amygdala’s involvement in emotion regulation, as indicated by several studies (3). Additionally, some research suggests a neuroimmunology explanation, mentioning how stress-related cascades in the brain may lead to the exacerbation of psychosocial factors in DBS patients, thereby contributing to the implementation of suicidality in this population (4).

Interestingly, suicide was observed both in successful and unsuccessful treatments throughout DBS: some studies circumscribed the rate of suicide only to premorbid risk factors such as a previous history of depression and treatment resistance, suggesting excluding the high suicide risk in DBS patients (5). Given the statistical prevalence of mood disorders affecting 60–70% of suicide victims, particularly post-hospital discharge, DBS implant recipients with prior depression or psychological fragility may face heightened suicide risk (6). A thorough pre-implant psychiatric assessment is crucial. Nevertheless, suicide is explicitly mentioned in the patient information leaflet of the Boston Scientific ® device for DBS implants (7). Vercise™ Genus™ DBS System: Indications). The stark contrast presented may instigate neuroethical debates, commencing with the therapeutic misconception inherent in DBS treatment, potentially encompassing the suicide risk (8). It underscores the imperative of meticulously apprising patients about this potential hazard. Moreover, there’s a need to recognize that informed consent may be susceptible to neurobiological biases, particularly in patients whose basal ganglia are altered because of neurodegenerative pathology, even before the permanent neuroanatomical implants of the DBS electrodes (9).

## 2 Case presentation

We describe the case of a 66-year-old Caucasian female, residing in Switzerland, with a history of Parkinson’s Disease. The patient, living at home with her husband, is now retired. She worked part-time in administrative roles until 2005. Following a gastrectomy in an oncological context in 2005, she was granted disability benefits.

The patient presented a condition evolving since 2015, marked by initial symptoms of rigidity and bradykinesia in the right upper limb. She has been diagnosed with Parkinson’s disease, reaching stage IV (Hoehn and Yahr) (10) with a predominant akineto-rigid tremor syndrome on the right side. Her primary status included frequent leg stiffness, pain in the left hemi body, and discomfort due to others’ gazes during dyskinesias. She also complains of intellectual slowness. Concentration difficulties during reading were found in a neuropsychological essay. Speech-related concerns were also evident, with the patient feeling misunderstood by her interlocutors and often asked to repeat herself. Graphomotor difficulties were also described as rendering her handwriting challenging to decipher. The patient presented a neurological follow-up consultation concerning her Parkinson’s disease following an earlier hospitalization for the initiation of an apomorphine pump, awaiting subsequent DBS surgery performed in 2019. A Semi-Structured Clinical Interview (Parkinson’s Disease Behavioral Scale) (11) performed before DBS implantation, revealed a sad mood without specific suicidal plans. The patient admits struggling to accept her illness and feeling ashamed. She described being easily worried and anxious, even about trivial matters.

The overall treatment history demonstrates a comprehensive approach with various medications and interventions to manage the evolving symptoms. Challenges in medication tolerance have been addressed through adjustments and changes in the treatment plan, including the utilization of an Apomorphine pump. After replacing Requip with Madopar® and adjusting the pump infusion rate, the patient currently reported an overall poor tolerance, experiencing an enduring OFF state throughout the day with internal tremors, generalized weakness, and accidental falls at night. Additionally, she noted more sporadic dyskinetic episodes, predominantly on the right side. Notably, the patient also reported the emergence of hallucinations, specifically auditory and sensing a person in her husband’s bed.

Diagnostic examinations, including DATSCAN in 2016 and brain MRI in 2022, revealed involvement of presynaptic dopaminergic pathways and an atrophic appearance of the cerebellar parenchyma, respectively. After symptom aggravation and the absence of significant progression after DBS treatment failure, the patient underwent a deeper differential diagnosis investigation. She gave her consent for inclusion in the National *RSMR Registry*, and the finally diagnosed condition was identified as *Multiple System Atrophy, Parkinsonian Type (ORPHA98933)*, classified as a subtype of a pathology (ICD-11 code 8D87.01) (12). The diagnostic journey commenced in 2016 when initial contact was made with a specialized center. A precise confirmation was documented in April 2023, following thorough clinical evaluation and imaging analysis (**Supplementary Figures 1**, **2**) (13).

Regarding the psychiatric history, she underwent a long psychotherapeutic course with a psychiatrist from 2005 to 2020 for an indefinite depressive state characterized by apathy and poor motivation. This ended during COVID-19-related restrictions, citing a reluctance to engage in remote consultations. Initially, the patient displayed hesitancy towards conventional antidepressant medication despite eventual compliance. She opted for alternative remedies like St. John’s Wort. Subsequently, she exhibited receptivity primarily towards mood-regulating interventions, notably engaging in psychotherapeutic modalities while maintaining a degree of reservation towards antidepressant pharmacotherapy. Her psychiatric follow-up commenced again in 2023 when seeking support for obtaining disability benefits after her gastric neoplasm diagnosis, leading to a diagnosis of recurrent depressive disorder. The patient was finally treated with an SSRI (Selective serotonin receptor inhibitor) antidepressant, escitalopram 20 mg 1x/day, and some benzodiazepine as an anxiolytic. The patient has no tobacco history and occasionally consumes alcohol. She also reported emotional lability, transitioning from laughter to tears, and feeling more emotionally sensitive than before. She had significantly isolated herself, limiting social interactions to text messages. She expressed significant discomfort related to her illness, feeling embarrassed and ashamed, and avoiding socializing with friends due to concerns about being seen in her condition with dyskinesias, clumsiness during meals, and tremors. The patient reported major depressive episodes with no psychotic symptoms, notably after the diagnosis of the rare disease confirmation in August 2023. On this occasion, she took her whole psychotropic treatment, intending to die. The patient asked for help once feeling sleepy, and her husband understood this episode as a consequence of the neurodegenerative aggravation of her illness. Her husband was finally not informed about the suicidal intention since the patient asked for maximal discretion with the doctors in the hospital, where she was briefly admitted for clinical observation. A primary ambulatorial psychotherapeutic course was activated, with a psychiatrist expert for the elderly age conducting a 60’ interview every two weeks directly at the home of the patient.

The patient was actively involved in parish activities until limitations related to her neurodegenerative disease compelled her to discontinue. Her daily life was circumscribed to watching television. Over the past two years, there has been a notable shift in her autonomy, with collaborative efforts between the patient and her husband in managing shopping, household affairs, and administrative tasks, with no external assistance.

### 2.1 Catamnesis

The presented clinical case report delineates the admission of a patient to the intensive care unit (ICU) consequent to benzodiazepine poisoning, specifically Rivotril® (clonazepam), with a Glasgow Coma Scale (GCS) of 3/15 upon ambulance arrival at her residence. Discovered unconscious in the bathroom by her spouse, who promptly summoned emergency assistance, the patient underwent toxicological analyses, affirming the presence of benzodiazepines in her urine screening. Upon admission to the ICU, a pump delivering the antidote flumazenil was activated to facilitate gradual arousal. After 72 hours, the antidote’s efficacy became apparent, and the patient regained consciousness. Subsequent to this, a neurologic assessment aimed at excluding organic etiologies associated with potential autonomic nervous system involvement was conducted. The patient admitted to deliberately consuming the entire benzodiazepine treatment, equivalent to 25 mg of clonazepam, with suicidal intent, withholding this intention from her husband, underscoring the need for discretion.

### 2.2 Psychiatric intervention

During an urgent psychiatric evaluation to assess suicide risk, the patient expressed alarm, conveying disappointment at the failure of her attempt and expressing a desire to devise new strategies, even within the hospital setting. Anxiolytic therapy with Seroquel ® (quetiapine) 25 mg three times daily was administered to stabilize mood, coupled with daily assessments by the psychiatric consultation team during her awakening in the ICU. Both pharmacological and psychotherapeutic interventions proved effective, as the patient began to value interactions with psychiatrists and sought an increase in consultations. She also articulated a profound need to express her suffering, noting the absence of prior inquiries into its underlying cause. The patient recounted her profoundly religious husband’s consistent hope for her potential recovery from her neurodegenerative illness, while she, convinced of her terminal state for several months, had contemplated suicide on multiple occasions. This episode is not the patient’s first demonstration of suicidal intentions, as evidenced by prior interventions from the psychiatric consultation team when she attempted suicide again, ingesting a combination of drugs, including sedatives and antidepressants, without success. However, on this occasion, she asserted having ingested a genuinely lethal dose (**Table 1**).

**Table 1:** Timeline.

Timeline	Event and interventions
Pre-Hospital	Patient suffering from a severe neurodegenerative disease found unconscious in the bathroom by spouse; ambulance called for first aid; Glasgow Coma Scale (GCS) of 3/15.
Admission	Toxicological analyses confirmed benzodiazepines Rivotril ® (clonazepam) in urine screening. Flumazenil pump activated.
Intensive Care Unit (72 hours)	Antidote took effect; patient regained consciousness and gradual awakening. Patient assessed daily by the psychiatric consultation team in ICU for suspected suicide attempt.
Post-Admission	• Neurologic evaluation: rule out organic dysautonomic causes. • Psychiatric suicidal assessment: Patient admitted to intentional consumption of entire benzodiazepine treatment (25 mg clonazepam) with suicidal intent. She expresses disappointment at failed suicide attempt, desires new strategies • Initiation of treatment and psychotherapeutic alliance Anxiolytic therapy with Seroquel ® (quetiapine) 25 mg thrice daily commenced. Patient did not disclose intentions to husband, stressing the need for discretion.

## 3 Discussion

This report documents the patient’s suicidal tendencies, revealing a state of moderate depression intertwined with reactive suicidal ideation related to her physical condition. A notable aspect is the emergence of a dependency dynamic with her husband and a loyalty conflict. Despite her desire to keep her actions secret, the patient does not hesitate to contemplate a second attempt.

The psychoanalytic assessment highlights the intricate interplay between her emotional struggles, relational dependencies, and existential beliefs. The patient’s inclination towards secrecy and the conflict of loyalty underscores the complexity of her psychological landscape. The decision to abstain from the procedure of assisted suicide demonstrates a nuanced negotiation between personal convictions and external factors, notably her husband’s religious stance. The disappointment with the DBS outcome appears to have intensified her existential disillusionment, revealing a profound need for understanding and addressing the intricacies of her emotional turmoil. The emergence of culpability in the plea for assisted suicide may be construed as the patient’s ultimate pursuit of internal autonomy and self-determination, disrupting the bonds of consent and cooperation with her family, who would otherwise validate her terminal state. The challenging role of a psychiatrist involved in the follow-up of DBS would be to include the option of assisted suicide before the worsening of the neurodegenerative disorder leads to apathy and reduction of their status to volition.

### 3.1 Patient perspective

The patient navigated through a complex web of emotions, marked by significant dissatisfaction and profound feelings of guilt towards her family, particularly her husband, whose unwavering optimism was fueled by strong religious convictions. This emotional burden intensified following her neurodegenerative diagnosis, manifesting both physically and psychologically. Physically, she experienced a gradual decline in her health, while psychologically, she grappled with the emergence of depressive symptoms, apathy, and diminished initiative. Despite her husband’s hopeful outlook on DBS therapy and medical advancements, the patient’s mood notably deteriorated following unsuccessful attempts.

The patient’s distress reached a critical juncture when the possibility of assisted suicide was broached during psychiatric consultations. Moreover, she found herself in a precarious position, lacking concrete protective factors due to physical limitations that impeded active engagement with life. Compounding her distress was the struggle to reconcile her desire for death with her religious beliefs, which prevented her from openly communicating her inner turmoil to her family.

This conflict culminated in severe depression punctuated by suicidal thoughts and two suicide attempts within a relatively brief period, despite no prior history of such behavior or emotional instability. The patient’s trust in antidepressant medication waned, leading to the exploration of alternative treatment options. Through collaboration with a liaison psychiatrist, she agreed to undergo thymic-regulatory treatment with quetiapine to address emotional fluctuations and moments of distress.

Despite her profound despair and disillusionment with science, which had failed to deliver the *“miraculous”* solution she had hoped for through DBS therapy, the patient ultimately refrained from considering assisted suicide, driven by her husband’s strong religious convictions. Paradoxically identifying as an atheist, her frustration stemmed from both the failure of medical interventions and the perceived betrayal of her family’s unwavering support and prayers. As she grappled with these conflicting emotions, the patient recognized the need for further exploration in psychotherapeutic sessions to unravel the layers of her distress and guide a comprehensive treatment plan that addresses her psychiatric and psychoanalytic needs.

### 3.2 Neuroethical analysis

This case presents several neuroethical challenges. These challenges stem from the interplay between her progressive neurological condition, the psychiatric implications, the treatment decisions, and the differing belief systems within her family unit. Neuroethical analysis requires a careful consideration of seven core ethical considerations. This analysis seeks to transcend mere identification of ethical issues by integrating international best practices to propose practical solutions, with a particular focus on managing treatment expectations within the therapeutic alliance.

First, autonomy and informed consent. The notion of autonomy is foundational to ethical clinical practice. Yet, as noted by Beauchamp and Childress, autonomy can be compromised in neurodegenerative diseases due to cognitive decline, rendering informed consent a process rather than a one-time event (14). In this context, the use of advance directives and the appointment of healthcare proxies becomes crucial to respecting the patient’s autonomy as the disease progresses (15). Nevertheless, it is important to acknowledge the limitations inherent in advance directives. While they offer a means for individuals to outline their treatment preferences in advance, they may not encompass every possible scenario or account for changes in medical technology and understanding. Additionally, the interpretation and application of these directives may pose challenges, particularly in situations where the patient’s current wishes diverge from those expressed in the past. Thus, while advance directives are valuable tools, they must be supplemented with ongoing communication and shared decision-making processes to ensure that the patient’s autonomy is respected and upheld to the greatest extent possible throughout the course of their illness. Furthermore, educational materials tailored to the patient’s cognitive level are essential for ensuring that the patient can make informed decisions about treatments such as DBS and participation in research registries (16).

Second, beneficence and non-maleficence. The notion of beneficence—to act in the patient’s best interest—is challenged by the patient’s worsening condition despite treatment, and the principle of non-maleficence—avoiding harm—is strained by the patient’s suicidal actions. The healthcare team must balance the pursuit of interventions that could potentially benefit her, like DBS, against the risk of exacerbating her suffering if these interventions fail.

The patient’s quality of life, marked by emotional lability and social withdrawal, invites a reconsideration of the effectiveness of current treatments in addressing the holistic needs of the individual. A multidisciplinary approach that includes psychological, social, and possibly spiritual support can help improve the patient’s quality of life (17). Social support groups can play a significant role in reducing feelings of isolation, and home-based care can help the patient feel more comfortable and dignified in managing her symptoms (18).

Third, privacy and confidentiality are also paramount, yet they must be navigated carefully when the safety of the patient is at stake (19). The patient’s request for maximal discretion regarding her suicidal attempt poses a dilemma. Clinicians must balance the patient’s right to confidentiality with the need to share information to ensure her safety (20). While respecting patient confidentiality is a core tenet, it must be balanced with the need for transparent communication with family members who are integral to the patient’s support system, especially when safety is a concern. Family meetings, held with the patient’s consent, can provide a forum to discuss these issues and to determine the extent of information sharing necessary for providing comprehensive care (21).

Fifth, cultural and religious sensitivity. The patient’s atheistic views contrast with her husband’s religious beliefs, creating an ethical dilemma regarding respect for differing values and the impact on treatment choices. This cultural and religious sensitivity requires a nuanced approach to care that respects both the patient’s and the family’s beliefs and values.

Sixth, end-of-life decision-making. The discussion around assisted suicide, although ultimately not pursued by the patient, raises significant ethical questions. It challenges the healthcare team to consider the legal, moral, and compassionate dimensions of such an option. This ethical challenge is amplified in jurisdictions where, unlike Switzerland, assisted suicide is legally sanctioned.

Finally, a particularly challenging ethical issue is the management of treatment expectations. Neurodegenerative diseases often involve treatments that promise significant improvement but may fail to deliver expected results, leading to psychological distress. It is the ethical responsibility of clinicians to ensure that patients have a realistic understanding of their disease and the potential outcomes of treatment, adhering to the principles of beneficence and nonmaleficence (14). Continuous dialogue about the course of the disease and the benefits and limitations of treatments such as DBS is essential in this regard.

Clinicians must navigate the delicate balance between fostering hope and providing an honest prognosis. The importance of managing expectations ethically cannot be overstated, as unrealistic expectations can lead to worse clinical outcomes and diminished trust in the therapeutic relationship (22). Therefore, avoiding to unrealistically present DBS or other neurotechnological intervention as a panacea or miraculous solution is paramount to respect the personal autonomy of patients and ensure a trustworthy doctor-patient relationship. Regular assessment of the patient’s cognitive abilities and shared decision-making can ensure that the patient’s understanding is aligned with the reality of her condition (23). Furthermore, when ethical dilemmas arise, consultation with hospital ethics committees can offer valuable insights.

## 4 Conclusions

The case of a 66-year-old woman highlights the intricate interaction between neurodegenerative disease, psychiatric challenges, and socio-religious dynamics. Initially diagnosed with Parkinson’s disease, her condition progressed to Multiple System Atrophy, Parkinsonian Type, unresponsive to conventional treatments. This led to severe psychological distress, including depression and suicidal thoughts, exacerbated by a conflict between her atheism and her husband’s religious optimism. While psychiatric interventions like quetiapine and psychotherapy stabilized her mood, persistent suicide attempts underscored deep-seated hopelessness. A nuanced approach considering her psychiatric state, autonomy, and family dynamics is crucial. Psychoanalytical exploration uncovered feelings of guilt and disillusionment with medical science. Establishing a therapeutic alliance provided some stability, yet existential struggles persist. This case underscores the importance of a comprehensive, interdisciplinary treatment plan addressing biological, emotional, and social factors. Effective communication and shared decision-making, especially regarding complex family beliefs and patient autonomy, are essential in managing chronic illnesses. Priorities include enhancing quality of life, addressing depressive symptoms, ensuring safety, and adapting care to evolving needs.

## Data availability statement

The original contributions presented in the study are included in the article/**Supplementary Material**. Further inquiries can be directed to the corresponding authors.

## Ethics statement

The studies involving humans were approved by Hôpitaux Universitaires de Genève. The studies were conducted in accordance with the local legislation and institutional requirements. The participant provided their written informed consent to participate in this study. Written informed consent was obtained from the individual for the publication of any potentially identifiable images or data included in this article.

## Author contributions

AD’I: Writing – review & editing, Writing – original draft, Visualization, Project administration, Methodology, Investigation, Formal analysis, Data curation, Conceptualization. MI: Writing – review & editing, Writing – original draft, Validation, Supervision, Resources, Project administration, Funding acquisition, Conceptualization.
