# Corrigendum: Deep brain stimulation and suicide attempts in treatment-resistant patients: a case report and neuroethical analysis

**DOI:** 10.3389/fpsyt.2025.1619883

**Published:** 2025-05-15

**Authors:** Ambra D’Imperio, Marcello Ienca

**Affiliations:** ^1^ Département de Psychiatrie, Hôpitaux Universitaires de Genève, Geneva, Switzerland; ^2^ Institut für Geschichte und Ethik der Medizin, Technische Universität München, Munich, Germany; ^3^ College of Humanities, École Polytechnique Fédérale de Lausanne (EPFL), Lausanne, Switzerland

**Keywords:** DBS (deep brain stimulation), suicidality, neuroethical considerations, multiple system atrophy Parkinsonian predominant type, case report

In the published article, there was an error in affiliations. Affiliation 2 read as: “Institut für Geschichte und Ethik der Medizin, Technische Universität München, Munich, Switzerland”. The affiliation should have been written as “Institut für Geschichte und Ethik der Medizin, Technische Universität München, Munich, Germany”.

The authors apologize for this error and state that this does not change the scientific conclusions of the article in any way. The original article has been updated.

