# Supplementary figures and images for: Deep brain stimulation and suicide attempts in treatment-resistant patients: a case report and neuroethical analysis
Source: Front Psychiatry. 2024 Jun 26;15:1398777. doi: 10.3389/fpsyt.2024.1398777 (PMC11234500; doi:10.3389/fpsyt.2024.1398777)

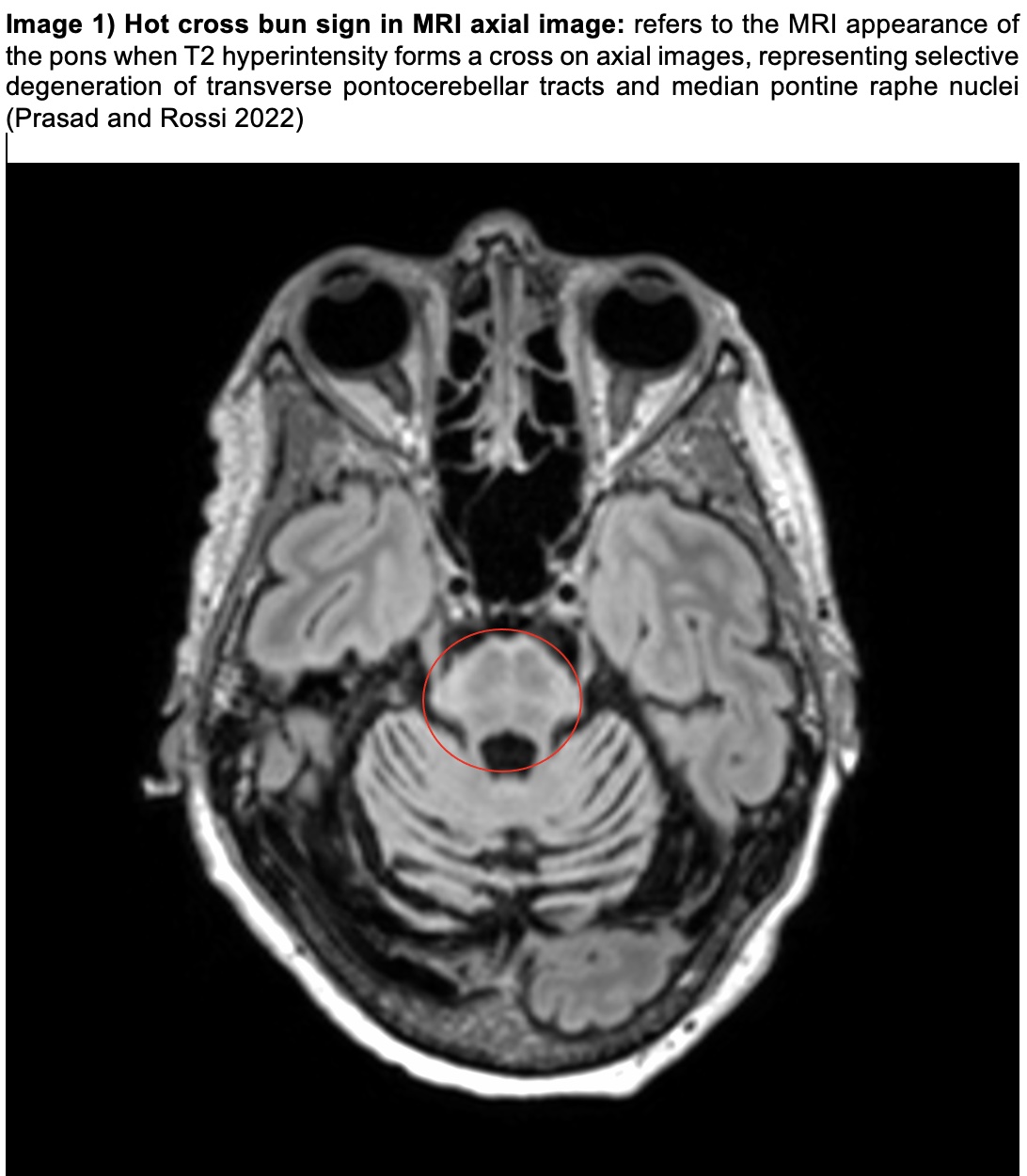

Supplement: Supplementary file 1 [file Image_1.jpeg]

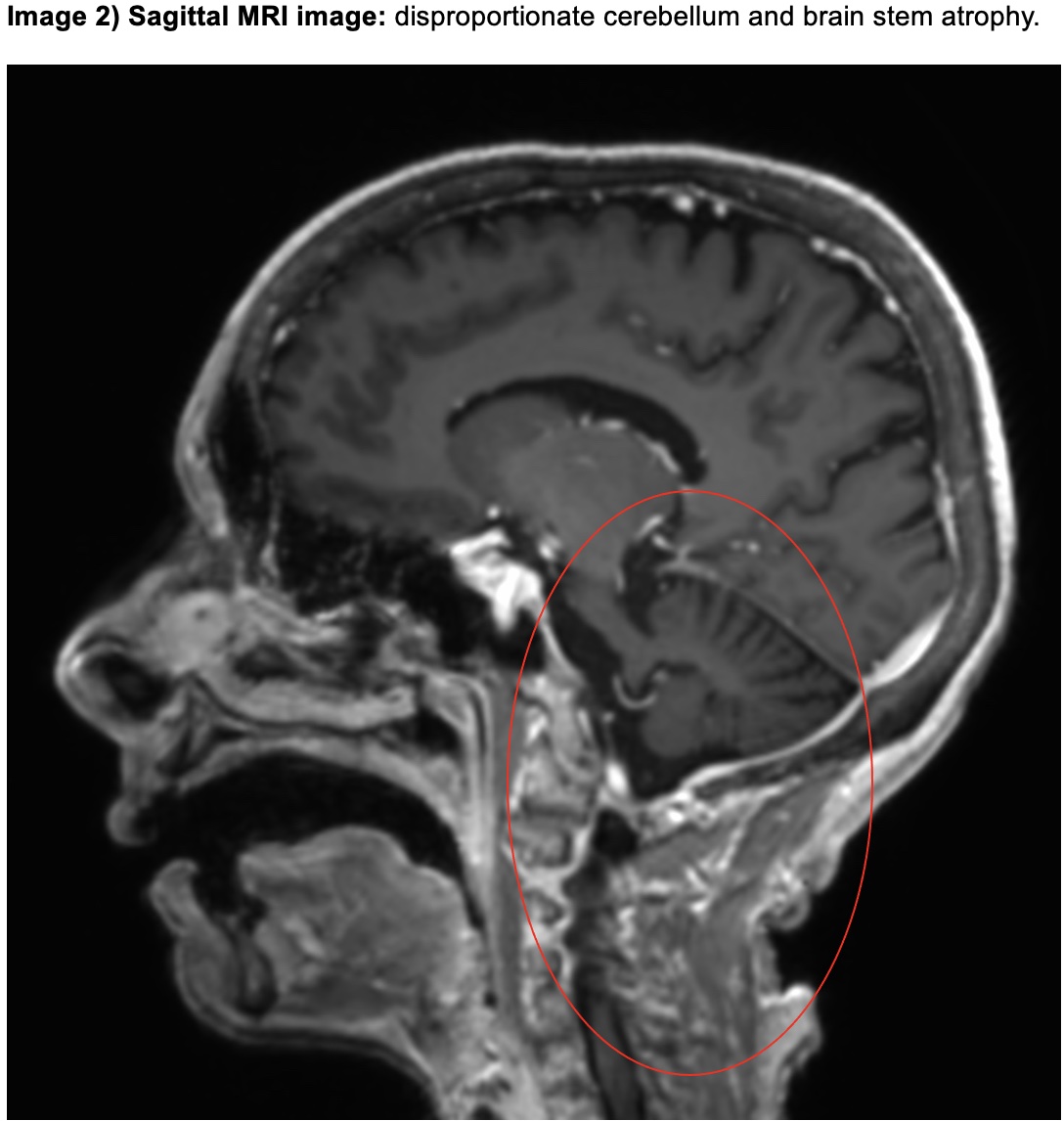

Supplement: Supplementary file 2 [file Image_2.jpeg]
